# Prenatal THC Does Not Affect Female Mesolimbic Dopaminergic System in Preadolescent Rats

**DOI:** 10.3390/ijms22041666

**Published:** 2021-02-07

**Authors:** Francesco Traccis, Valeria Serra, Claudia Sagheddu, Mauro Congiu, Pierluigi Saba, Gabriele Giua, Paola Devoto, Roberto Frau, Joseph Francois Cheer, Miriam Melis

**Affiliations:** 1 Department of Biomedical Sciences, Division of Neuroscience and Clinical Pharmacology, University of Cagliari, 09042 Monserrato, Italy; ftraccis@unica.it (F.T.); valeria.serra@unica.it (V.S.); claudiasagheddu@unica.it (C.S.); mauro.congiu@unil.ch (M.C.); p.saba@unica.it (P.S.); gabriele.giua@inserm.fr (G.G.); pdevoto@unica.it (P.D.); roberto.frau@unica.it (R.F.); 2 Department of Anatomy and Neurobiology, University of Maryland School of Medicine, Baltimore, MD 21201, USA; JCheer@som.umaryland.edu

**Keywords:** cannabis, behavior, dopamine, electrophysiology, neurodevelopment, neuropsychiatric disorders, sex

## Abstract

Cannabis use among pregnant women is increasing worldwide along with permissive sociocultural attitudes toward it. Prenatal cannabis exposure (PCE), however, is associated with adverse outcome among offspring, ranging from reduced birth weight to child psychopathology. We have previously shown that male rat offspring prenatally exposed to Δ9-tetrahydrocannabinol (THC), a rat model of PCE, exhibit extensive molecular, cellular, and synaptic changes in dopamine neurons of the ventral tegmental area (VTA), resulting in a susceptible mesolimbic dopamine system associated with a psychotic-like endophenotype. This phenotype only reveals itself upon a single exposure to THC in males but not females. Here, we characterized the impact of PCE on female behaviors and mesolimbic dopamine system function by combining in vivo single-unit extracellular recordings in anesthetized animals and ex vivo patch clamp recordings, along with neurochemical and behavioral analyses. We find that PCE female offspring do not show any spontaneous or THC-induced behavioral disease-relevant phenotypes. The THC-induced increase in dopamine levels in nucleus accumbens was reduced in PCE female offspring, even when VTA dopamine activity in vivo and ex vivo did not differ compared to control. These findings indicate that PCE impacts mesolimbic dopamine function and its related behavioral domains in a sex-dependent manner and warrant further investigations to decipher the mechanisms determining this sex-related protective effect from intrauterine THC exposure.

## 1. Introduction

The prevalence of mental disorders worldwide is 13% and, alarmingly, 50% of these are established before the age of 14 years, with most cases undetected and untreated [1,2]. Additionally, the average delay between symptom onset and initial treatment is of about 11 years [3], a delay that potentially jeopardizes clinical outcome. This highlights the relevance of an early identification of these disorders to design a timely therapeutic intervention.

Children with a parent who has a mental illness or substance use disorder have a higher risk of psychiatric problems themselves [4,5]. Critically, prenatal cannabis exposure (PCE) increases the risk for child psychopathology, ranging from affective symptoms to attention deficit hyperactive disorder (ADHD) and psychotic-like experiences [6,7,8,9,10,11]. With recreational cannabis legalization and permissive sociocultural attitudes expanding worldwide, and the use of cannabis among pregnant women on a sharp rise [12,13,14], concern has increased over the long-term negative impact on next-generation health (i.e., pediatric concern) [15,16,17,18,19]. Since the main psychoactive ingredient of cannabis, Δ9-tetrahydrocannabinol (THC), crosses the placenta and interferes with the endocannabinoid system, a signaling pathway key in proper neural development [20,21,22,23], it is plausible that PCE may be teratogenic [12]. PCE is thought to act as a “first hit” on neurodevelopmental trajectories [24]. Of note, a sex bias in response to PCE is well recognized [6,7,8]. However, the underlying mechanisms are not sufficiently studied because causal inference is difficult to be established in population studies. Additionally, gender as a variable in the susceptibility to the consequences of cannabis exposure on neurocognitive and behavioral development of the offspring is seldom examined [25]. Thus far, insights into the mechanisms of how sex interacts with PCE to generate unique effects in the progeny can be solely gained from preclinical studies.

Animal models of PCE show that male exposed offspring are more susceptible than female offspring to dysfunctions in cognitive processing and emotional regulation [26,27,28,29,30,31,32,33,34,35]. Thus, female sex often is a protective factor in response to same intrauterine environmental insults. However, many individual (e.g., species, strain, age) and experimental (e.g., design, drug, dosage, route, regimen) variables along with objective endpoints (e.g., behavioral paradigm, experimental technique) might influence sex dimorphism. For instance, we have shown that only male PCE offspring exhibit, at pre-puberty, a psychotic-like endophenotype [34]. This is accompanied by extensive molecular and synaptic changes in dopaminergic neurons of ventral tegmental area (VTA), converging on a mesolimbic hyperdopaminergic state susceptible to either THC or stress [34,36]. However, whether female offspring display a different disease-relevant behavioral phenotype likely associated with yet unidentified alterations in mesolimbic dopamine system function remains unknown. Here we show that many of the detrimental effects induced by PCE on male dopamine neurons and their neurochemical and behavioral readout are absent in females at pre-puberty. Of note, PCE female progeny manifest a risk-safer phenotype along with normal social behavior and adaptive coping strategies to acute stress. Collectively, these data extend our understanding of the multifaceted developmental effects imposed by PCE on the rat mesolimbic dopamine system and warrant further investigations to decipher the mechanisms determining the process that confers a sex-specific resistance against prenatal THC.

## 2. Results

### 2.1. Impact of PCE on Behavior in Female Preadolescent Rats

Evidence suggests a sex bias in response to PCE [6,7,8]. In particular, we found that male PCE rats display a psychotic-like phenotype in response to acute THC or stress and a higher sensitivity to a dopamine D2 agonist, and they engage in risk-taking behaviors [34,37]. However, female progeny does not exhibit sensorimotor gating deficit in response to PCE or acute THC [34]. Therefore, we first evaluated whether female progeny display other features of PCE male cohort.

**Figure 1:**
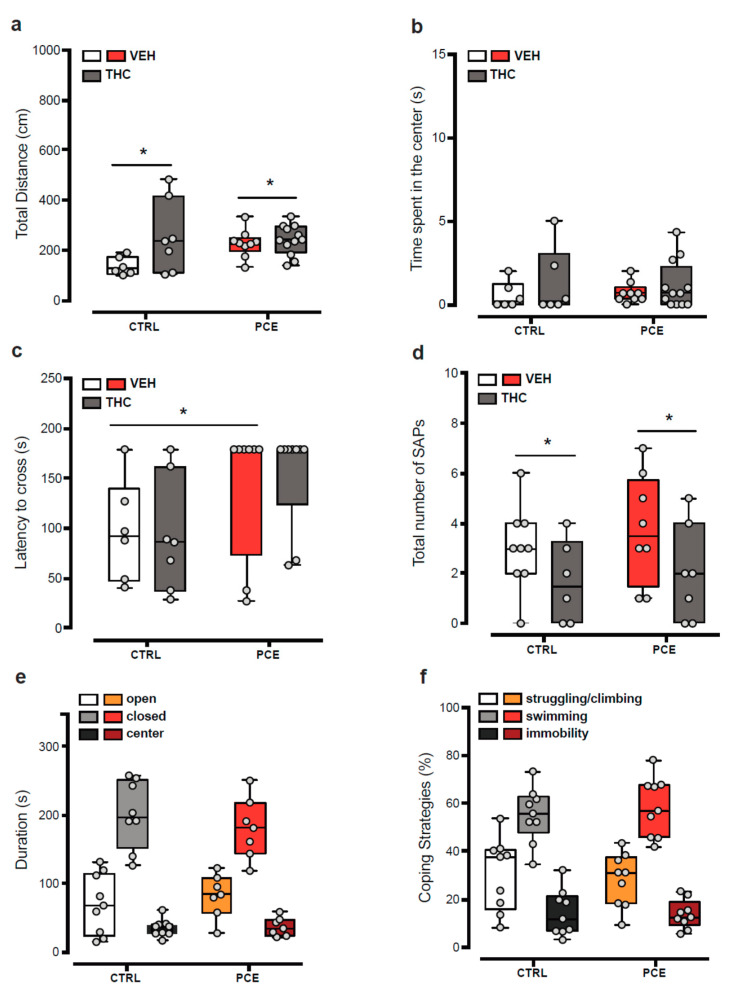
PCE effect on female preadolescent offspring behavior. (**a**) Spontaneous locomotor activity in female offspring, measured as total distance traveled in a novel open-field arena. Dose of Δ9-tetrahydrocannabinol (THC) is 2.5 mg–1 kg (s.c.) and was administered 15 min before the test (*n* = 6–12/group). (**b**) Time spent (s) in the center of the open-field arena. THC or vehicle (VEH) administration does not affect the thigmotaxic behavior of prenatal cannabis exposure (PCE) females in comparison to CTRL. (**c**) The latency to crossing the wire-beam bridge is increased by PCE (*n* = 7–9). (**d**) THC challenge decreases the number of stretched-attend postures (SAP) in both PCE and CTRL offspring during the wire-beam bridge test. (**e**) Behavioral responses in the elevated plus maze. No differences were found in the total duration of time spent by offspring in the closed, open arms, and center position (*n* = 7–9/group). (**f**) Effect of forced swim tests (FST) on coping strategies engaged by PCE and CTRL offspring (*n* = 9/group). Time spent (%) in struggling/climbing, active swimming, and immobility during the FST. All data are represented as box-and-whisker plots with single values (min to max). * *p* < 0.05.

When we measured spontaneous or THC-induced locomotor activity in vehicle (CTRL) and PCE female offspring in an open field arena, no differences were observed at baseline (two-way ANOVA, main effect of PCE: F(1,30) = 1.67, *p* = 0.206) and following an acute administration of THC (2.5 mg/Kg, s.c.; Figure 1a; two-way ANOVA, interaction, F(1,30) = 3.336, *p* = 0.1; main effect of THC: F(1,30) = 5.384, *p* = 0.027). Accordingly, no differences were observed in thigmotaxis between groups (Figure 1b; two-way ANOVA, interaction PCE × THC: F(1,29) = 0.091, *p* = 0.7645). Next, we investigated risk propensity by using the wire-beam bridge task, which evaluates a risk-taking phenotype in rodents by measuring their proclivity to cross a flexible bridge suspended over a 150 cm deep gap. PCE increased the latency to cross the wire-beam bridge (Figure 1c; two-way ANOVA, main effect of PCE: F(1,26) = 6.306, *p* = 0.018), but did not affect the response to acute THC (main effect of THC: F(1,26) = 0.031, *p* = 0.859) or the number of stretched-attend postures (Figure 1d; two-way ANOVA, main effect of THC: F(1,26) = 4.987, *p* = 0.034; interaction PCE × THC: F(1,26) = 0.091, *p* = 0.76). Since the spontaneous prudent phenotype observed in PCE females could be secondary to an anxiety-like state, we carried out the elevated plus maze test. In agreement with stretched-attend posture phenotypes (as index of anxiety-like phenotype) observed in the bridge test, we found no differences between the groups (Figure 1e; two-way ANOVA, interaction: F(2,41) = 0.776, *p* = 0.467). Finally, given that PCE male progeny do not adopt copying strategies in response to acute stressors [37], we evaluated the effects of forced swim test, as an acute inescapable stressor, on female offspring. PCE does not alter the amount of time spent in engaging active (struggling/climbing and swimming) or passive (immobility) strategies (Figure 1f; two-way ANOVA, interaction: F(2,48) = 0.293; *p* = 0.7475). Collectively, these results indicate that PCE does not induce in female offspring a susceptible phenotype resembling their male counterparts.

**Figure 2:**
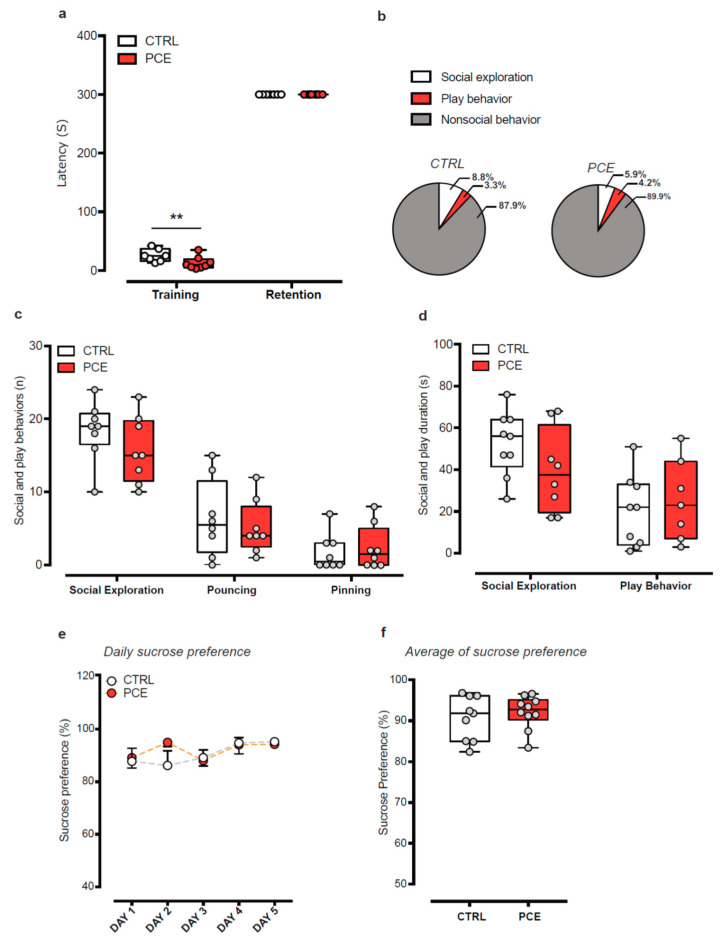
PCE impact on emotional memory, social interaction, and anhedonia-like behavior in female preadolescent rats.(**a**) Passive avoidance (PA) latency (s) to enter in dark compartment during training (before foot shock) and retention (24 h after foot shock) sessions (*n* = 7–8/group). (**b**) Pie chart representation of social (exploration and play behavior) and nonsocial behaviors exhibited during the social interaction test. No differences were found in terms of (**c**) number of social approaches, pouching and pinning and (**d**) total duration of time spent in social exploration and play behaviors (*n* = 7–9/group). (**e**) Daily sucrose preference (%) during the 5 days of sucrose preference testing and (**f**) in the average sucrose preference. Data are represented as average ± s.e.m. (for sucrose preference curves) or as box-and-whisker plots with single values (min to max). **, *p* < 0.01.

We next considered the possibility that PCE might induce different disease-relevant phenotypes in female progeny. Thus, we investigated whether PCE female offspring might exhibit three traits of maladaptive behaviors that occur in several psychiatric disorders, the avoidance of aversive or threatening stimulus, the social behaviors, and the ability to feel pleasure toward positive emotional states. We measured the passive avoidance latency during the training session and 24 h later (i.e., retention): although an effect was observed during the training session (Figure 2a; two-way ANOVA, Bonferroni’s multiple comparisons test CTRL vs. PCE, *p* = 0.006), no differences were found during the retention session (Figure 2a; two-way ANOVA, Bonferroni’s multiple comparisons test, CTRL vs. PCE, *p* > 0.999). We then assessed whether PCE affected their social capabilities, but found no differences between the groups, in terms of duration of both nonsocial and social activities during social interaction test (Figure 2b; two-way ANOVA, interaction: F(2,42) = 1.789, *p* = 0.179). Specifically, PCE did not alter the frequency and the duration of social exploration, measured as sniffing approaches to the partner (Figure 2b,c; frequency: unpaired *t*-test, t15 = 1.322, *p* = 0.205; duration: t15 = 1.52, *p* = 0.149). Accordingly, no differences were found in the frequency of pinning and pouncing (Figure 2c; unpaired *t*-test, pinning: t14 = 0.355, *p* = 0.72; pouncing: t14 = 0.550, *p* = 0.59) and in the total time spent in playing behaviors (Figure 2d; t14 = 0.606, *p* = 0.55).

Finally, we investigated whether PCE affected the animal ability to experience pleasure by performing the sucrose preference test (SPT), whose changes are suggestive of anhedonia, a cardinal symptom in depressive states. PCE did not affect female offspring daily sucrose preference (Figure 2e; RM two-way ANOVA, F(4,83) = 0.863, *p* = 0.4893) either at baseline (day 1) and during the subsequent days (from day 2 to 5). Accordingly, no differences were found in the average preference for sucrose solution (Figure 2f; unpaired *t*-test, t17 = 0.664, *p* = 0.51). Altogether, PCE does not impact any of the behaviors studied here in females.

### 2.2. PCE Effect on Mesolimbic Dopamine Transmission

**Figure 3:**
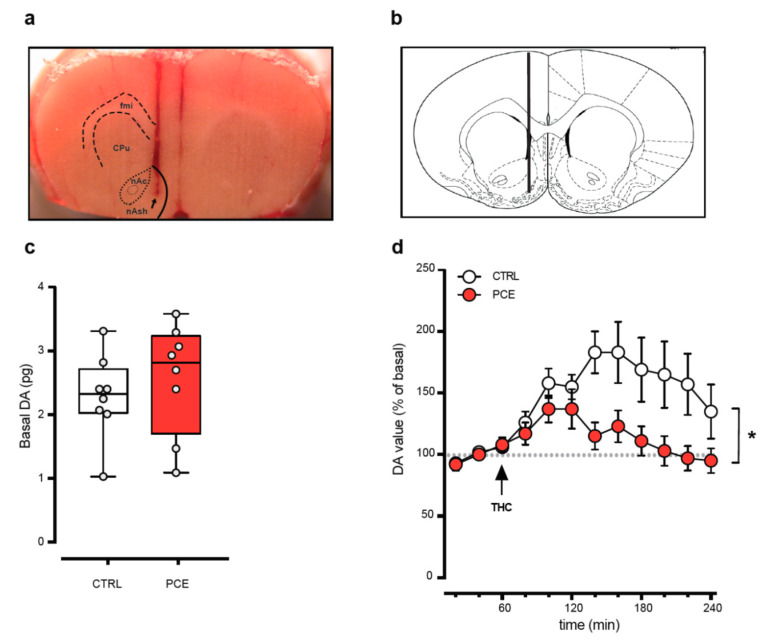
PCE impact on THC-induced increase of accumbal DA level in female preadolescent offspring. (**a**) Representative photograph of microdialysis probe location into the nucleus accumbens shell. The arrow indicates the tip of the probe. Abbreviations: fmi, forceps minor corpus callosum; CPu, caudate putamen; nAc, nucleus accumbens core; nAsh, nucleus accumbens shell. (**b**) Schematic representation of cerebral area targeted by the probe as indicated by the vertical line (nAsh, AP: +1.5, L: ±0.7, V: −7.0 from bregma) (**c**) Basal dopamine (DA) values (pg) measured in the nAsh (*n* = 7–8/group). Data are expressed as pg/sample (the box-and-whisker plot shows min to max with single values). (**d**) Time course of the effect of acute THC (2.5 mg/kg i.p.) on extracellular DA levels in nAsh (*n* = 7–8/group). Data are shown as percentage of baseline and are represented as mean ± s.e.m. *, *p* < 0.05.

To elucidate whether the apparent protection from PCE impact observed in female progeny may be related to sex differences in the effects of PCE on mesolimbic dopamine system function, we next carried out cerebral microdialysis experiments in the target region of the shell of nucleus accumbens (NAcS) (Figure 3a,b). In behaving preadolescent female offspring, we found no alteration in basal extracellular dopamine levels between CTRL (2.29 ± 0.23 pg) and PCE (2.56 ± 0.30 pg) females (Figure 2c; unpaired *t*-test, t14 = 0.72, *p* = 0.48). Notably, in PCE offspring, THC-induced increase in extracellular dopamine was smaller compared to CTRL (Figure 3d; RM two-way ANOVA, PCE: F(1,13) = 5.11, *p* = 0.04).

**Figure 4:**
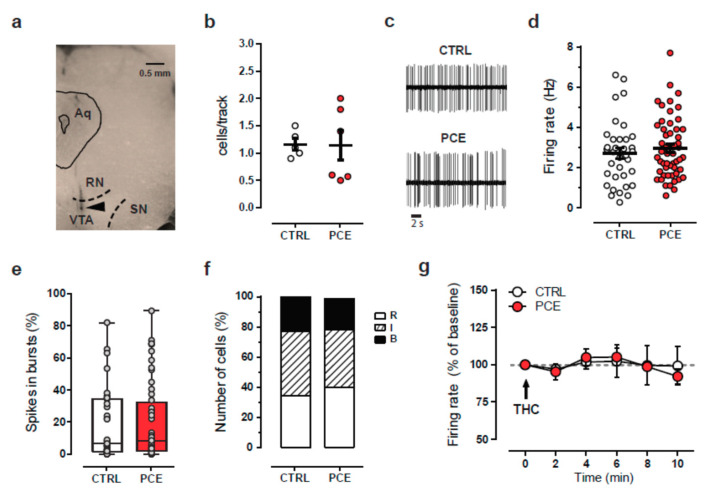
PCE effect on electrophysiological properties of putative dopamine neurons recorded in vivo from female preadolescent rats. (**a**) Coronal midbrain section from a preadolescent female rat showing the recording site (black triangle) in the ventral tegmental area. Abbreviations: Aq, aqueduct; RN, red nucleus; SN, substantia nigra, VTA, ventral tegmental area. (**b**) Scatter plot showing the average number of spontaneously active VTA dopamine neurons encountered per track. PCE (*n*_rats_ = 6) CTRL (*n*_rats_ = 5). (**c**) Representative traces of spontaneous firing activity of dopamine neurons from female offspring. (**d**) Spontaneous firing frequency of VTA dopamine cells from CTRL and PCE female rats. Data are represented as means s.e.m. with single values. (**e**) Percentage of spikes in burst displayed by dopamine neurons in female offspring. Data are represented as a box-and-whisker plot with single values, min to max. (**f**) The stack bars represent the percentage of dopamine cells displaying different firing patterns: R = regular; I = irregular; B = bursty. (**g**) Time course of the effect of acute THC (0.5 mg/kg, i.v) on the firing frequency of putative VTA dopamine neurons from PCE (*n*_cells_ = 5) and CTRL (*n*_cells_ = 5). Data represented as average ± s.e.m.

Next, we investigated whether PCE affects electrophysiological properties of VTA dopamine neurons (Figure 4a) putatively projecting to NAcS by performing single-unit extracellular recordings in vivo. In anesthetized rats, neither the number of spontaneously active cells (Figure 4b; unpaired *t*-test, t9 = 0.05; CTRL: 1.16 ± 0.11, *n* = 5; PCE: 1.15 ± 0.27, *n* = 6) nor the average firing frequency (Figure 4c,d; unpaired *t*-test, t85 = 0.71; CTRL: 2.71 ± 0.27, *n* = 35; PCE: 2.96 ± 0.21, *n* = 52) differ in PCE (*n*_rats_ = 6, *n*_cells_ = 52) compared with CTRL (*n*_rats_ = 5, *n*_cells_ = 35). Similarly, the firing mode, as expressed by the percentage of spikes in bursts (Figure 4e; unpaired *t*-test, t85 = 0.45) and pattern of activity (Figure 4f; PCE regular, *n*_cells_ = 21; irregular, *n*_cells_ = 20; bursting, *n*_cells_ = 11; CTRL regular, *n*_cells_ = 12; irregular, *n*_cells_ = 15; bursting, *n*_cells_ = 8), did not change between the groups (chi-square test = 0.33). We next examined whether dopamine neurons responded differently to the effect of an acute THC challenge alike males [37]. We found that THC (0.5 mg/kg i.v.) did not alter the firing frequency of VTA dopamine neurons in either group of preadolescent females (*n*_cells_ = 5 in both groups; Figure 4g; RM two-way ANOVA, F(5,40) = 0.28).

### 2.3. Intrinsic and Synaptic Properties of Putative VTA Dopamine Neurons in PCE Females

**Figure 5:**
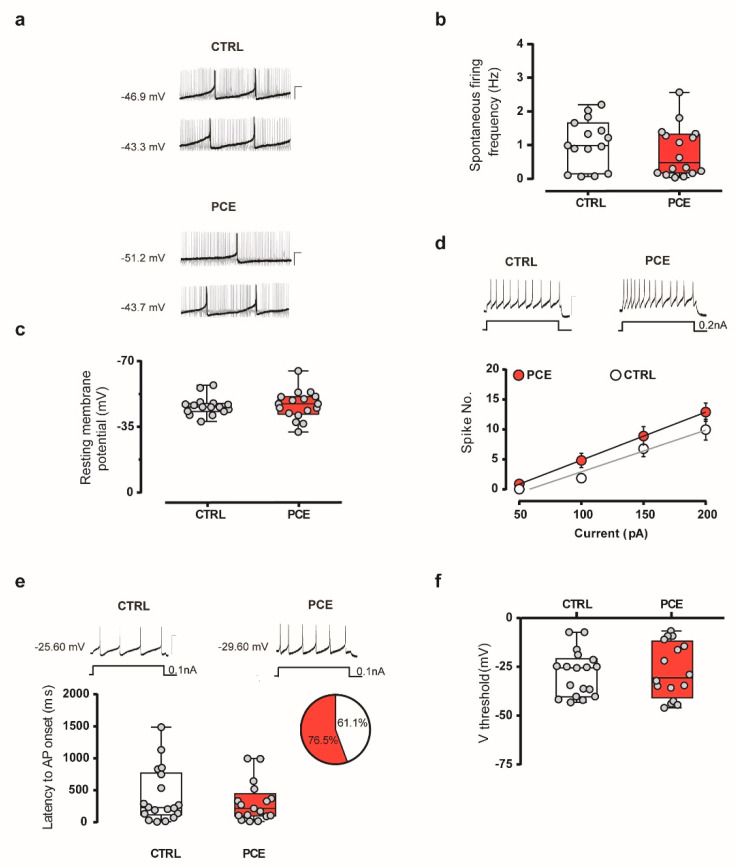
Intrinsic properties of putative VTA dopamine neurons are not affected by PCE in female preadolescent offspring. (**a**) Representative traces of spontaneous activity of dopamine neurons recorded from acute VTA slices from CTRL and PCE offspring (*n*: 15 and 16 experiments from CTRL and PCE slices, respectively). Calibration bars, 100 ms, 20 mV. (**b**,**c**) PCE (*n*_cells_ = 16–17, *n*_rats_ = 11/group) does not affect the spontaneous firing frequency (**b**) and resting membrane potential (**c**) compared with CTRL cells (*n*_cells_ = 15–16, *n*_rats_ = 7/group). (**d**) Spike frequency in response to somatically injected current does not differ between CTRL (*n*_cells_ = 18, *n*_rats_ = 7/group) and PCE (*n*_cells_ = 17, *n*_rats_ = 11/group) offspring. Insets show representative traces of evoked action potentials (APs) in response to maximum current injected. Calibration bar, 200 ms, 100 mV. Data are represented as average values per animal ± s.e.m. (**e**) Top: representative traces of evoked APs in response to somatic current injection of 0.1 nA. Calibration bar, 100 ms, 50 mV. Bottom: latency to the first AP of dopamine neurons elicited in response to current injection of 0.1 nA is not modified by PCE. Inset shows a pie chart graph with the proportion of cells eliciting APs at 0.1 nA current (CTRL in white, PCE in red). (**f**) Threshold of AP elicited by depolarizing current does not vary between PCE (*n*_cells_ = 17, *n*_rats_ = 11/group) and CTRL (*n*_cells_ = 18, *n*_rats_ = 7/group) groups. Unless otherwise specified, all data are represented as box-and-whisker plots with single values (min to max).

In male offspring, PCE imposes changes in both intrinsic and synaptic properties of putative dopamine neurons of the VTA by inducing a hyperexcitable phenotype [34]. Ergo, we performed whole-cell patch clamp recordings from putative dopamine neurons of the lateral portion of the posterior VTA in females. In acute brain slices, PCE did not impact VTA dopamine cell spontaneous firing rate (Figure 5a,b; unpaired *t*-test, t29 = 0.984, *p* = 0.333) and their resting membrane potential (Figure 5c; unpaired *t*-test, t31 = 0.218, *p* = 0.282). PCE did not change spike frequency in response to somatically injected currents (Figure 5d; linear regression, F(1,124) = 1.757, *p* = 0.187) and the latency to the first action potential (AP) elicited by the smallest current injected able to reliably elicit APs (Figure 5e; unpaired *t*-test, t33 = 0.765, *p* = 0.449). No differences were also found in the voltage threshold of AP elicited by a depolarizing current between groups (Figure 5f; t33 = 0.577, *p* = 0.567).

**Figure 6:**
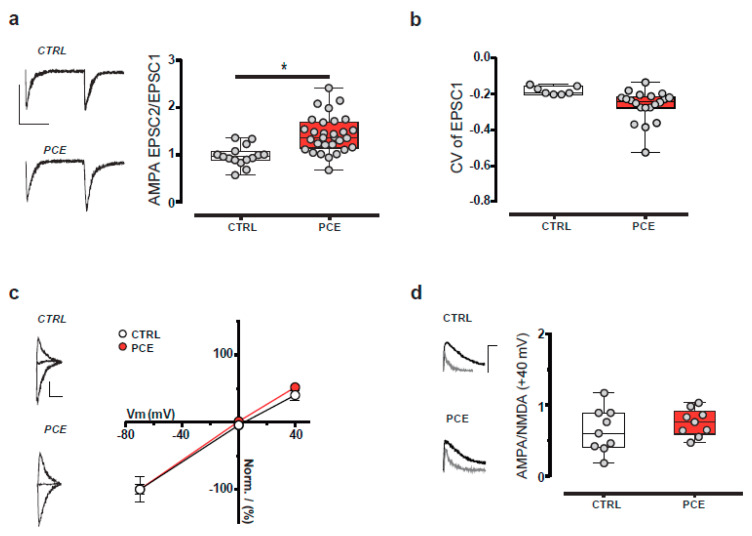
Excitatory synaptic properties of putative VTA dopamine neurons are not changed by PCE in female preadolescent offspring. (**a**) Dopamine cells from PCE (*n*_cells_: 30, *n*_rats_: 11/group) offspring exhibit an increased paired-pulse ratio (EPSC2/EPSC1) of AMPA EPSCs compared to CTRL (*n*_cells_: 15, *n*_rats_: 7/group) group. The left-hand panel shows representative traces of paired AMPA EPSCs recorded from VTA putative dopamine neurons of CTRL and PCE offspring. Calibration bar: 25 ms, 50 pA. (**b**) PCE effects on 1/CV2 values (PCE *n*_cells_: 20, CTRL *n*_cells_: 7). (**c**) Current–voltage relationship (*I*–*V*) curves of AMPA EPSCs recorded from dopamine neurons in PCE (*n*_cells_: 10, *n*_rats_: 12/group) and CTRL (*n*_cells_: 8, *n*_rats_: 8/group) offspring. Data are represented as mean ± s.e.m. AMPA EPSCs traces recorded at −70 mV, 0 mV, and +40 mV from CTRL and PCE rats are shown on the left. Calibration bar: 10 ms, 25 pA. (**d**) The AMPA/NMDA ratio is not affected by PCE. The left-hand panel shows representative traces of AMPA and NMDA EPSCs traces recorded from dopamine neurons held at +40 mV in slices from PCE (*n*_cells_: 9, *n*_rats_: 9/group) and CTRL (*n*_cells_: 9, *n*_rats_: 7/group) offspring. Calibration bar: 10 ms, 50 pA. Unless otherwise specified, all data are represented as box-and-whisker plots with single values (min to max). *, *p* < 0.05.

We next examined the synaptic properties of dopamine neurons by applying paired-pulse (50 ms interstimulus interval) modulation protocol. PCE facilitated the paired-pulse ratio (PPR) of α-amino-3-hydroxy-5-methyl-4-isoxazolepropionic acid receptor (AMPAR)-mediated excitatory postsynaptic currents (EPSCs) (Figure 6a; unpaired *t*-test, t43 = 2.681, *p* = 0.010) without changing their coefficient of variation (CV) (Figure 6b; unpaired *t*-test, t25 = 1.463, *p* = 0.156). No differences in the current–voltage relationship of AMPA EPSCs (Figure 6c; linear regression, F(1,50) = 0.343, *p* = 0.561) and in the AMPA/NMDA ratio were found between groups (Figure 6d; unpaired *t*-test, t16 = 0.922, *p* = 0.370). Collectively, these findings indicate that PCE does not affect intrinsic properties and postsynaptic responsiveness to the glutamatergic transmission of VTA dopamine neurons of female offspring. Nonetheless, we cannot rule out that PCE might anatomically and functionally affect the cell subpopulations of the VTA other than dopaminergic ones.

## 3. Discussion

The present study reveals a protection of preadolescent female progeny to prenatal THC exposure. Specifically, our findings show that not only does PCE not render females susceptible to acute THC [34], but that females show a resilient phenotype associated with normal mesolimbic dopamine (DA) system function. Specifically, PCE does not impact female offspring ability to cope with acute stress, to experience pleasure, and to learn avoiding an unpleasant stimulus, thus broadening the spectrum of potentially disease-relevant behaviors examined that PCE could have impacted.

Our findings support and extend previous animal studies where sex-specific differences in the effects of in utero exposure to THC, and more generally to cannabinoids, have been described [32,35,38,39,40,41]. Remarkably, despite the importance of examining both sexes, this gap has only been partially recognized in research, but not yet fully addressed as analyzing only one sex, and pooling the data from both sexes is still a common practice. Nonetheless, evidence points to the impact of PCE being a function of sex despite the variables of species, strain, and age studied. On the one hand, the detrimental effects of PCE on male sexual motivation, social interaction, spatial cognition, and sensorimotor gating functions associated with alterations in the function of cortical and midbrain regions have been described [32,34,35,37,40,42]. On the other hand, in females, PCE affects spontaneous locomotion in adulthood [38] along with their motivation for food and morphine, an effect ascribed to changes in their mesolimbic DA activity [41]. These contrasting findings arise from the many differences in experimental conditions among the studies but underline that sex-specific responses to PCE during fetal life do exist and depend on differential effects on pathways and brain regions.

Our data, obtained during the prepuberal window of vulnerability, support previous studies examining different ages and showing that PCE does not affect female socioemotional behavior and coping strategies to acute stress, but decreases the reactivity of mesolimbic DA system as measured by the changes of extracellular DA levels in the nucleus accumbens shell (NAcS) in response to an acute challenge of THC. This latter, while in sharp contrast to our findings in male PCE counterparts [34], would be in agreement with the data showing, in adult PCE female Wistar rats, a reduction in the levels of 3,4-dihydroxyphenylacetic acid (DOPAC)/DA ratio in both NAcS and VTA homogenates [41]. The DOPAC/DA ratio is a measure of DA turnover and has long been ascribed to mirror the activity of DA neurons in the VTA and the integrity of DA system function [43]. However, when we examined the DOPAC/DA ratio in our preadolescent PCE-behaving Sprague Dawley female rats, we did not observe either PCE or THC effects (data not shown). These discrepancies could be due to different strains of rats used, the age of the animals, or the preparation for neurochemical analysis (behaving animals vs. brain region homogenates). Additionally, our finding that the firing frequency of VTA DA cells is not altered by PCE, as indexed by our in vivo and ex vivo recordings, and that acute THC does not modify DA neuron spontaneous activity in vivo, supports the notion that axonal DA release is not linearly scaling with somatic action potential firing [44,45]. Although the latter is usually regarded as a proxy for DA signaling and its behavioral readout, many other factors regulate DA release in the target region, and at many stages [46], including DA production and vesicular loading, action potential propagation, regulation of DA reuptake, to name a few. To complicate this issue, sex differences in the mechanisms regulating DA release in the target regions are yet to be discovered. Finally, and importantly, sex dimorphisms in the mechanisms involved in neuronal intrinsic excitability and synaptic plasticity are seldom examined [32,47], especially before puberty; this is remarkable since sex hormones contribute to the differentiation of developmental trajectories and to dynamic changes in endocannabinoid signaling from adolescence to adulthood [48].

The observation of protection in females to the deleterious effects of PCE at pre-puberty supports the hypothesis that male sex is a risk factor for discrete neuropsychiatric disorders of developmental origin [49,50,51,52,53]. One could speculate that because the growth rate of male fetuses is faster than females in the womb, the risk of undernutrition in males is increased. Notably, undernutrition is a contributing factor for the development of non-communicable diseases later in life [54]. Fetal growth differences are usually associated with sex dimorphisms in placental expression of genes relevant for coping abilities to adverse in utero environment [55,56]. Although no differences in weight gained by PCE offspring compared to controls at pre-puberty in both females (data not shown) and males were found [34], symmetrical fetal growth restriction, with a catch-up growth by post-natal day 21, and placental dysfunction have been reported [57].

Alternatively, one could argue that sex differences associated with global transcriptomic profiles and neuroprotective effects of glial cells in females [58,59,60] might protect them from the same environmental insult, i.e., THC. It is noteworthy that the increased tightness of the blood–brain barrier in female neonatal rats [60] might protect them by reducing brain disposition of THC (or its metabolites), and the resulting interference with endocannabinoid system during fetal neurodevelopment. In particular, two xenobiotic transporters (i.e., Abcb1 and Abcg2) are involved in brain disposition of THC [61], and their expression shows sex dimorphisms in both the brain and the placenta [62]. Indeed, female placentas express higher levels of Abcg2 mRNA than males [62], a transporter key in transplacental pharmacokinetics and fetal protection [63] that ensures proper function of fetoplacental unit throughout pregnancy [64]. Since phytocannabinoids, including THC, inhibit the Abcg2 [65], one could speculate that brain THC concentrations might be higher in males than females, thus limiting its teratogenic impact on female neurodevelopment.

Collectively, our findings show that the outcome of PCE in the female mesolimbic dopamine system differs from that seen in males, though it might not strictly be a protected version of the male outcome. Further investigations are needed to uncover potential PCE effects on females that might be region- and circuit-specific and associated with a disease-relevant phenotype, potentially mutually exclusive. Finally, our results highlight the importance of consistently examining the mechanisms underlying physiological and pathological states in both sexes to develop tailored and personalized therapies.

## 4. Materials and Methods

### 4.1. Subjects

All experimental procedures were carried out according to the European legislation EU Directive 2010/63 and were approved by the Animal Ethics Committees of the University of Cagliari and by Italian Ministry of Health (auth. no. 256/2020). We made all efforts to minimize pain and suffering and to reduce the number of animals used.

Primiparous female Sprague Dawley rats (Envigo) were used as mothers and single-housed during pregnancy. Offspring were weaned at postnatal day (PND) 21 and were housed in a climate-controlled animal room (21 ± 1 °C; 60% humidity) under a normal 12 h light–dark cycle (lights on at 7:00 a.m.) with ab libitum access to water and food. Because we previously found that PCE is a risk factor for psychotic-like endophenotype only in male progenies [34], the present investigation aimed at testing whether female offspring shows a different disease-relevant behavioral phenotype linked to alterations in mesolimbic dopamine system function. Thus, all the experiments were conducted in female rats during preadolescence (PND15–28). All animals included in this study underwent a single pharmacological manipulation. To control for litter effects, no more than two females were used from each litter for the same experiment. To minimize the total number of animals used for the study, all the additional female pups in each litter were used for other experiments.

### 4.2. Drugs and Treatments

Δ9-Tetrahydrocannabinol (THC) resin was purchased from THC PHARM GmbH (Frankfurt, Germany) and dissolved in ethanol at 20% final concentration. Then, THC was suspended in a vehicle (VEH) solution containing 1–2% Tween^®^ 80 and diluted with sterile saline (0.9% NaCl).

To model PCE, rat dams were administered subcutaneously (s.c.) with THC (2 mg kg^−1^, 2 mL kg^−1^) or VEH once per day from GD5 until GD20. This dose of THC was chosen because it does not elicit substantial behavioral responses or tolerance after repeated administration [66]. Moreover, it fails to affect maternal or non-maternal behavior, or offspring litter size [34]. Notably, this dose of THC is equivalent to the current estimates of moderate cannabis consumption in humans since it is similar to the THC content in mild joints (5%) [67].

### 4.3. Behavioral Tests

#### 4.3.1. Locomotor Activity

Thigmotaxis and motor behavior of female offspring were recorded for 40 min in a novel, transparent open activity cage (Omnitech Digiscan monitoring). Female offspring (PND 24–28) were placed in a novel, square open-field arena (42 cm × 42 cm) surrounded by four 40 cm, highly transparent Plexiglas walls, and locomotor activity was measured for 40 min using an Omnitech Digiscan monitoring system (Columbus, OH, USA) as indicated [34]. Each cage (42 × 42 × 40 cm^3^) had two sets of 16 photocells located at right angles to each other, projecting horizontal infrared beams 2.5 cm apart and 2 cm above the cage floor. After 15 min of VEH or THC (2.5 mg kg^−1^, s.c.) administration, the rats were placed in the center of the arena, and the total distance traveled and the time spent in the periphery and in the center of the arena were assessed.

#### 4.3.2. Wire-Beam Bridge Test

Risk propensity was evaluated using a variant of the wire-beam bridge task, which was modified for rats, as detailed [34]. Briefly, the apparatus consists of two Plexiglas platforms (156 cm high) connected by a horizontal, flexible wire-beam (100 cm long) bridge. One of the platforms was surmounted by a Plexiglas wall (52 cm high) placed right above the edge of the platform (start position). After 15 min of VEH or THC (2.5 mg kg^−1^, s.c.) administration, female offspring (PND 24–28) were individually placed in the start position and the entire session (3 min duration) was video-recorded. Behavioral measures included latency (s) to cross the bridge and reach the other platform and the number of stretch-attend postures (SAPs) exhibited by animals during the test.

#### 4.3.3. Elevated Plus Maze

The test was performed as previously described [34], under dim (10 lux) light. Briefly, the apparatus was made of black Plexiglas with a dark blue floor and consisted of two opposing open arms (length of 40 cm, width of 9 cm) and two closed arms (wall height of 15 cm), which extended from a central square platform (9 × 9 cm^2^), positioned 70 cm high from the ground. Animals were tested after an acclimation period of 2 days in the experimental room. At the testing day, female rats (PND 24–28) were individually placed on the central platform facing the open arm. The entire sessions were video-recorded for 5 min and later scored by blinded observers. Behavioral measures included the time spent and entries into each partition of the maze. An arm entry was counted when all the four paws were inside the arm.

#### 4.3.4. Forced Swim Test

To evaluate their responsiveness to an acute inescapable stressor, female offspring were tested by using a modified Porsolt forced swim test (FST) [68], as previously indicated [37]. The rats (PND 24-28) were individually placed into a transparent cylinder (50 × 20 × 20 cm^3^) filled with 2 L of cold water for 10 min. During the test, the animals were video-recorded and later scored by blinded observers. Behavioral measures included passive coping (measured as duration of time spent floating with the absence of any movement except those necessary to keep the nose above water) and active coping behaviors (time spent swimming and climbing/struggling).

#### 4.3.5. Passive Avoidance

Passive avoidance (PA) was tested in a two-way shuttle box using an experimental protocol modified for rats [69]. The apparatus consisted of two equal-size (35 × 35 cm^2^) compartments: light (white and illuminated with 24 V–10 W bulb) and dark (black and dark) divided by a manually operated sliding door at the floor level. The dark compartment was equipped with an 18-bar insulated shock grid connected to a shocker. On day 1 (training session), female rats (PND 24–28) were individually placed in the light compartment facing away from the door and let to explore the chamber for 5 min. The latency to enter into the dark compartment was measured. When the animal completely entered into the dark compartment, the sliding door was closed and a 0.5 mA shock was delivered for 2 s. After 30 s, the animal was removed from the apparatus and returned to its home cage. The animals that did not enter the dark compartment within 5 min were excluded from the analysis. After 24 h (retention session), each animal was placed into the light chamber and the latency to enter the dark compartment was recorded to a maximum of 5 min.

#### 4.3.6. Social Interaction Test

Social interaction was tested as previously described [70,71]. Female rats (PND 24–26) were individually placed into a neutral, unfamiliar Makrolon cage (20 × 35 cm^2^) together with a weight- and age-matched female conspecific (born from a separate litter) for 10 min. During the test, the animals were video-recorded and later scored by blinded observers. Behavioral measures included the total duration and frequency of social exploration (i.e., sniffing approaches directed to any part of the body of the partner), social play fighting behavior (i.e., pouncing, pinning, boxing, wrestling, or chasing), and nonsocial behaviors (i.e., exploratory activities directed to environment, grooming, or digging activities).

#### 4.3.7. Sucrose Preference Test

Anhedonia was measured by using a modified protocol of sucrose preference test (SPT) [72]. Before the test, female offspring were habituated to the presence of two drinking bottles for 2 days after weaning. Then (PND 23), the rats were individually housed in a home cage with two drinking bottles—one containing 1% sucrose solution and the other tap water—for 5 days. The locations of the bottles were switched daily to reduce habituation bias. Experimental measures included sucrose preference as percentage (%) and averaged over the 5 days of testing. Sucrose preference was calculated as a percentage of the volume of sucrose intake over the total volume of fluid intake.

### 4.4. Cerebral Microdialysis

For cerebral microdialysis experiments, female rats were anesthetized with Equithesin and stereotaxically implanted with in-house-constructed vertical microdialysis probes (AN 69-HF membrane, Hospal-Dasco; cut-off 40,000 Dalton, 3 mm dialyzing membrane length) in the nucleus accumbens shell (from bregma: anterior–posterior: +1.5; lateral: ±0.7; ventral: −7.0) [72]. The day after probe implantation, artificial cerebrospinal fluid solution (ACSF; 147 mM NaCl, 4 mM KCl, 1.5 mM CaCl_2_, 1 mM MgCl_2_, pH 6–6.5) was pumped through the dialysis probes at a constant rate of 1.1 μL min^−1^ via a CMA/100 microinjection pump (Carnegie Medicine, Stockholm, Sweden) in freely moving animals. Samples were collected every 20 min and analyzed for dopamine content by high-performance liquid chromatography with electrochemical detection, as previously described [73]. When a stable baseline was obtained (three consecutive samples with a variance not exceeding 15%), THC (2.5 mg/kg, 2 mL/kg) was intraperitoneally (i.p.) administered, and sample collection continued for 2 h. On completion of the experiments, the rats were killed with an Equithesin overdose, and the brains were removed and sectioned using a cryostat (Leica CM3050 S) into 40 μm thick coronal slices to verify the anatomical locations of dialysis probes.

### 4.5. In Vivo Single-Unit Electrophysiological Recordings

PND 25–29 female offspring were anaesthetized with chloral hydrate (400 mg/kg i.p.) and were cannulated in their femoral vein for intravenous administration of drugs. The animals were placed in the stereotaxic apparatus, with their body temperature maintained at 37 ± 1 °C by a heating pad. Single-unit activity was extracellularly recorded with glass micropipettes filled with 2% Pontamine sky blue dissolved in 0.5 M sodium acetate from lateral posterior VTA (anterior–posterior −4.5 to −5.2 mm from bregma, medial–lateral 0.4–0.6 mm, and ventral −6.5 to −7.5 mm from surface). Putative dopamine neurons were isolated and identified according to well-established electrophysiological criteria such as their firing rate (<10 Hz), and duration of action potential (>2.5 ms). For each neuron, the firing rate (calculated as the total number of spikes occurring over time) and bursting activity (defined as the occurrence of two spikes at inter-spike interval < 80 ms and terminated when the inter-spike interval > 160 ms) were analyzed. In a subset of experiments, THC 0.5 mg/kg/mL was administered intravenously. At the end of the recording sessions, a 15 mA current was passed for 15 min through the micropipette to mark the position of the electrode within the recording site [37].

### 4.6. Ex Vivo Electrophysiological Recordings

The preparation of posterior VTA slices was performed as previously described [34]. Briefly, a block of tissue containing the midbrain was obtained from female offspring deeply anesthetized with isoflurane and the tissue sliced in the horizontal plane (300 µm) with a vibratome (Leica) in ice-cold low-Ca^2+^ solution containing the following (in mM): 126 NaCl, 1.6 KCl, 1.2 NaH_2_PO_4_, 1.2 MgCl_2_, 0.625 CaCl_2_, 18 NaHCO_3_, and 11 glucose. The slices were transferred to a holding chamber with ACSF (36–37 °C) saturated with 95% O_2_ and 5% CO_2_ containing the following (in mM): 126 NaCl, 1.6 KCl, 1.2 NaH_2_PO_4_, 1.2 MgCl_2_, 2.4 CaCl_2_, 18 NaHCO_3_, and 11 glucose. The slices were allowed to recover for at least 1 h before being placed, as hemislices, in the recording chamber and superfused with ACSF (36–37 °C) saturated with 95% O_2_ and 5% CO_2_. Cells were visualized using an upright microscope with infrared illumination (Axioskop FS 2 plus, Zeiss), and whole-cell patch-clamp recordings were made using an Axopatch 200B amplifier (Molecular Devices). The recordings were carried out in the lateral portion of the posterior VTA.

Current-clamp recordings were made with electrodes filled with a solution containing the following (in mM): 144 KCl, 10 HEPES buffer, 3.45 BAPTA, 1 CaCl_2_, 2.5 Mg_2_ATP, and 0.25 Mg_2_GTP, pH 7.2–7.4, 275–285 mOsm. As previously described, this solution had no effect on the holding current of the dopamine cells. Current-clamp experiments were performed in the absence of any pharmacological blocker, that is, in regular ACSF. Voltage-clamp recordings of the evoked EPSCs were made with electrodes filled with a solution containing the following (in mM): 117 cesium methanesulfonic acid, 20 HEPES, 0.4 EGTA, 2.8 NaCl, 5 TEA-Cl, 0.1 mM spermine, 2.5 Mg_2_ATP, and 0.25 Mg_2_GTP, pH 7.2–7.4, 275–285 mOsm. Picrotoxin (100 μM) was added to the ACSF to block GABAA-receptor-mediated inhibitory postsynaptic currents (IPSCs). Series and input resistance were monitored continuously online with a 5 mV depolarizing step (25 ms). Experiments were begun only after series resistance had stabilized (typically 10–30 MΩ), which was monitored by a hyperpolarizing step of −5 mV at each sweep every 10 s. Data were excluded when the resistance changed >20%. The data were filtered at 2 kHz, digitized at 10 kHz, and collected online with acquisition software (pClamp 10.2, Molecular Devices).

Dopamine neurons from the lateral portion of the posterior VTA were identified according to previously published criteria [34] as follows: cell morphology and anatomical location (that is, medial to the medial terminal nucleus of the accessory optic tract); slow pacemaker-like firing rate (<5 Hz); long action potential duration (>2 ms); and the presence of a large hyperpolarization-activated current (Ih > 100 pA) [74], which was assayed immediately after break-in using 13 incremental 10 mV hyperpolarizing steps (250 ms) from a holding potential of −70 mV. A bipolar, stainless steel stimulating electrode (FHC) was placed ∼100–200 μm rostral to the recording electrode and was used to stimulate at a frequency of 0.1 Hz.

The paired-pulse ratio (PPR), with an interstimulus interval of 50 ms, was calculated as the ratio between the second and the first postsynaptic currents (EPSC2/EPSC1) and averaged over 5 min. NMDA EPSCs were evoked while holding cells at +40 mV. The AMPA EPSC was isolated after bath application of the NMDA antagonist D-2-amino-5-phosphonovaleric acid (D-AP5, 100 µM). The NMDA EPSC was obtained by digital subtraction of the AMPA EPSC from the dual (AMPA + NMDA-mediated) EPSC [34].

### 4.7. Statistical Analysis

Statistical analysis was performed with GraphPad Prism 6 (San Diego CA, USA) software. Data from behavioral, microdialysis, and in vivo electrophysiological experiments were analyzed using two-tailed unpaired *t*-test or two-way ANOVA (followed by Bonferroni’s multiple comparisons test) when appropriated. Electrophysiological data were analyzed using Student’s *t*-test or linear regression when appropriate. The sample size was computed based on power calculations. The analysis assumptions are power = 0.9 and alpha = 0.5. Statistical outliers were identified with Grubb’s test (α = 0.05) and excluded from the analysis. Significance level was set at *p* < 0.05.

## Data Availability

The data presented in this study are available on request from the corresponding author.
